# Modelling size constraints on carbonate platform formation in groundwater upwelling zones

**DOI:** 10.1038/s41598-018-35771-z

**Published:** 2018-11-29

**Authors:** Mark N. Keppel, Vincent E. A. Post, Andrew J. Love, Adrian D. Werner, Jonathan D. A. Clarke, Todd Halihan

**Affiliations:** 1 Government of South Australia, Department for Environment and Water, 81–95 Waymouth Street, Adelaide, South Australia 5000 Australia; 2 National Centre for Groundwater Research and Training, and College of Science & Engineering, Flinders University, GPO Box 2100, Adelaide, South Australia 5001 Australia; 3 The Mars Society Australia, PO Box 327, Clifton Hill, Victoria, 3068 Australia; 4 Boone Pickens School of Geology, Oklahoma State University, 105 Noble Research Centre, Stillwater, Oklahoma 74078 USA

## Abstract

Carbonate depositional systems related to groundwater upwelling are ubiquitous around the world and form ecologically and culturally important features of many landscapes. Spring carbonate deposits record past climatic and hydrological conditions. The reconstruction of past processes using spring carbonate proxies requires fundamental understanding of the factors that control their geometry. In this work, we show that the spatial extent of spring carbonate platforms is amenable to quantitative prediction by simulating the early growth stage of their formation for the iconic mound springs in the central Australian outback. We exploit their well-defined, circular geometry to demonstrate the existence of two size-limiting regimes: one controlled by the spring flow rate and the other by the concentration of lattice ions. Deviations between modelled and observed size metrics are attributable to diminishing spring flow rates since formation, enabling assessment of the relative vulnerability of springs to further hydrological change.

## Introduction

**Figure 1:**
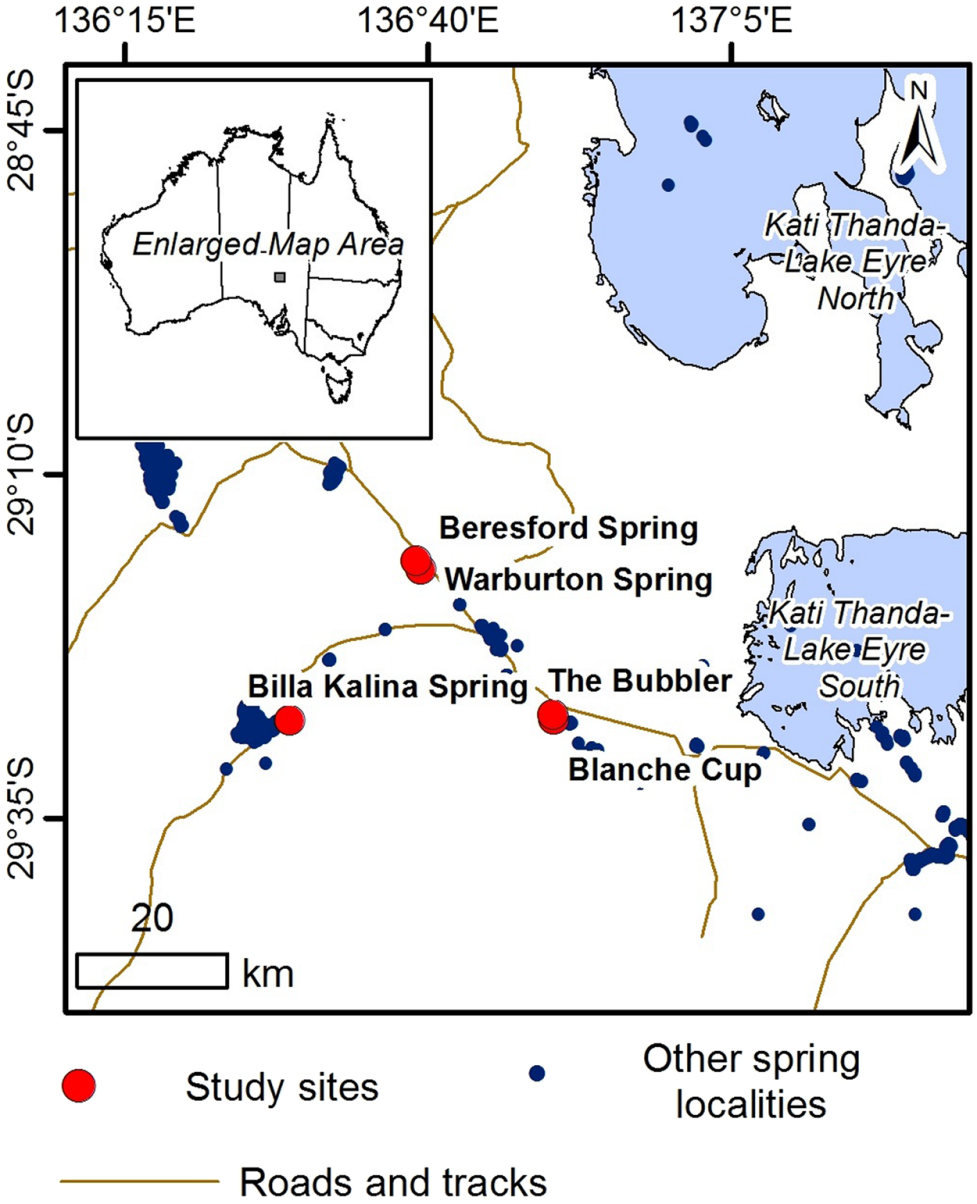
Location map of study sites. Inset show location in Australia.

Stable oxygen and carbon isotope analysis in combination with dating techniques of fossiliferous carbonate deposits in groundwater upwelling zones have previously been used to derive changes in climate and the onset of arid periods, as well as information about the long-term temporal dynamics of groundwater upwelling^1–7^. With the exception of some works that have modelled the shape of actively-forming depositional features^8–11^, most previous studies concerning the geochemical and sedimentological controls on spring carbonate deposition are field-based, with a particular emphasis on understanding carbonate precipitation triggers^12^. The principal controls on the morphology of carbonate mounds have been assumed to be either the availability of lattice ions^13^ or flow rates^14,15^. In this study, we used reactive transport modelling, i.e., numerical modelling of water flow and concomitant chemical reactions, to investigate the principal controls on the size of carbonate mounds associated with springs in the Kati Thanda-Lake Eyre South region of South Australia (Fig. 1). The springs in this region sustain unique wetland ecosystems and are of great cultural significance^15,16^; consequently, methodologies that provide insight into their life cycle are needed for their management and to aid conservation efforts.

Mound formation is a three-part process consisting of: (i) initial spring and wetland formation, (ii) mound growth and (iii) growth cessation and stabilisation. Kinetically-controlled CO_2_ degassing from emergent spring water as water flows away from the spring vent is central to this mound spring conceptual model. After a certain distance, calcium carbonate (CaCO_3_) precipitation, triggered by CO_2_ degassing, results in the build-up of a mound barrage that forms an enclosure for a central pool. The time required for sufficient degassing to trigger CaCO_3_ precipitation determines both the size of the central pool and surrounding mound. The CO_2_ degassing rate is dependent on flow turbulence and the surface area exposure of water to the atmosphere. Once a pool has formed, the degassing rate slows as the mound height increases and the water trapped in the central pool deepens and becomes less turbulent. Mounds reach maturity when the capacity of emergent spring water trapped within the spring pool changes from one of net precipitation to net dissolution and erosion, eventually leading to the development of a tail gutter through which water escapes to form a wetland delta at the mound base, to which carbonate precipitation shifts.

This model not only explains many of the morphological and geological features of carbonate mound springs found in the Kati Thanda-Lake Eyre South region, but also the great ages of mounds, which may reach tens of thousands of years^16^ relative to the comparatively fast precipitation rates found in the field^12^. Consequently, the early growth stage discussed in this paper describes a relatively short period of time in the life of a mound spring, but nevertheless an important one as it can provide a snapshot of the possible hydrological and hydrogeological conditions of a spring wetland and supporting aquifer at a given point in time.

Modelling past spring environments involves making assumptions about past hydrological conditions. Therefore, rather than calibrating a model to simulate observed mound sizes, this study’s objective was to test if, given the most defensible set of assumptions of past flow conditions, a reactive transport model can provide: (i) an explanation for the observed large variation in mound radii, and (ii) quantitative support for our proposed conceptual model of mound formation^12^.

**Figure 2:**
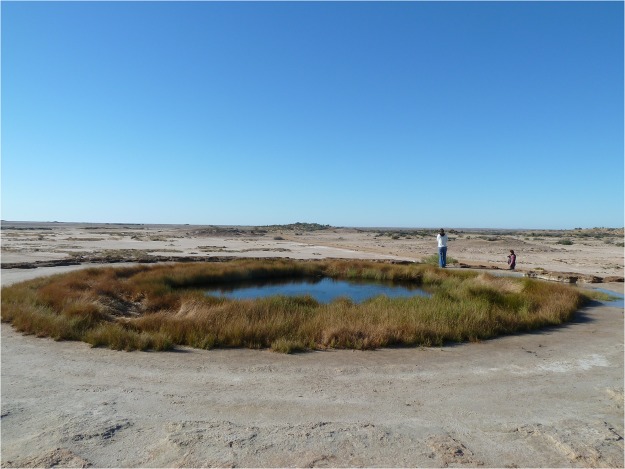
Photograph of a calcium carbonate mound at Blanche Cup, displaying dome-shaped, circular geometry (Courtesy G. Green).

**Figure 3:**
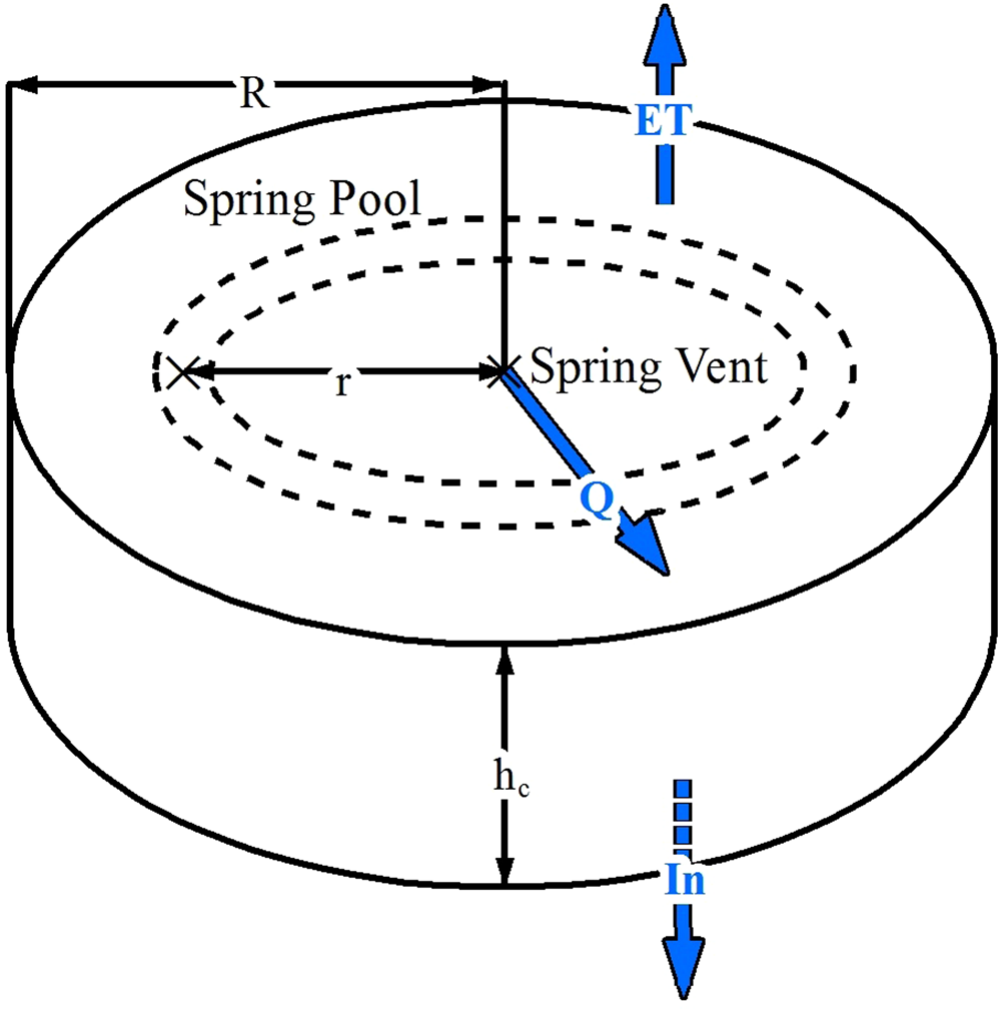
Graphical representation of the geometry and hydrological processes adopted for the modelling. R is the maximum radius of the spring wetland, r is the radial distance from the spring vent, Q is the spring discharge, ET and In are water-loss through evaporation and infiltration respectively, and h_c_ is the mean height of the water column. The dashed lines indicate the perimeter of one of multiple cells in the model with the cross indicating the midpoint where concentrations are calculated.

The studied pools, which are near-circular in shape, have a surface area between 100 and 420 m^2^, and associated well-vegetated wetlands. The mounds surrounding them are generally dome-shaped or shield-like (Fig. 2), with heights varying from 2 to 5 m. Their near-circular shape suggests that they initially formed by carbonate precipitation from discharging groundwater that flowed away from the spring vent in a radial fashion. This assumption is justified by the flat topography of the terrain. Consequently, the geometry of the earliest stage surface manifestation of a mound spring was conceptualised as a circular wetland (Fig. 3), with the discharging water radially flowing away from the vent.

Two end-member regimes are proposed to aid understanding of the factors limiting mound size. The first possibility is that the maximum radius of the mound is commensurate with the maximum distance that emergent spring water can flow away from the vent, which is limited by water loss in the surrounding wetland by evapotranspiration and infiltration. The second possibility is that the lattice ions (calcium and carbonate) that build the mound become depleted before the maximum migratory distance of water is reached, resulting in a mound radius smaller than the spring-supported wetland. For both regimes, a characteristic timescale exists. The timescale for the water-limited regime, *t*_*w*_, can be defined as the time required for the water to reach the edge of the wetland. The maximum extent of the circular wetland was based on water balance calculations, using measured spring discharges, as well as evapotranspiration and infiltration rates^13^ (See Methods). As the calculated flow velocity becomes almost negligible towards the edge of the wetland, the *t*_*w*_ was practically defined as the time require to reach 95% of the wetland radius.

Changes in the water composition in the modern spring wetland in the study region have been successfully simulated previously by a reactive transport model that includes degassing of dissolved CO_2_ and calcium carbonate precipitation^13^. With this model, a characteristic timescale for the lattice-ion limited regime can be determined. Here, this timescale, denoted *t*_*c*_, was taken as the time after which negligible calcium carbonate precipitation occurs (see Methods). The carbonate precipitation rate is a complex function of the composition and pH of the spring water, which vary as degassing and carbonate precipitation reactions progress; the characteristic timescale must therefore be evaluated numerically.

The analysis of timescales provides fundamental insight into the key factors that limit the extent of the spring mounds, but it does not provide any spatial or temporal constraints on the size of the mounds. Therefore, the current study also undertook reactive transport modelling to test if the size of the carbonate mound footprint, as well as the footprint of the central spring pool can be predicted based on knowledge of the spring discharge, water loss by evaporation and infiltration, and spring water composition. Consistent with the analysis of characteristic timescales, simulations considered CO_2_ degassing and CaCO_3_ precipitation, but within a spatially-explicit framework of radial-symmetric flow with water loss via evapotranspiration and infiltration. Such insights into the controls on carbonate mound morphology gained from the current investigation will also impact our understanding the palaeohydrology of the wetland and the supplying aquifer and therefore develop also our appreciation of the relative vulnerability of these wetlands and the associated groundwater resource to changes, both natural and anthropological.

## Results

**Figure 4:**
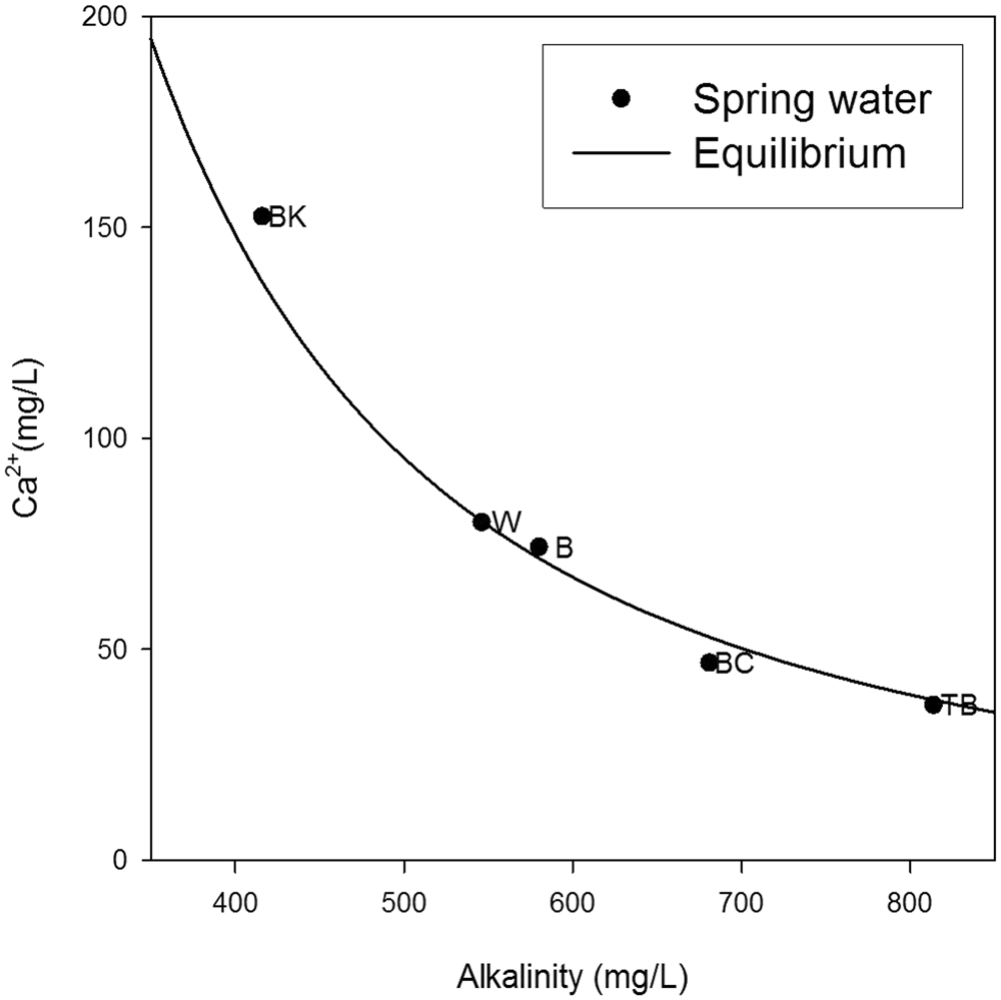
Plot of Alkalinity (mg/L as CaCO_3_) versus Ca^2+^ (mg/L) for emergent spring waters. The line represents groundwater, with an ionic strength of 0.07, in equilibrium with calcite at a CO_2_ partial gas pressure of 10^−1.5^ atm. B Beresford, BC: Blanche Cup, BK: Billa Kalina, TB: The Bubbler, W: Warburton.

Spatial measurements taken from sampled springs in the Kati Thanda-Lake Eyre South region of South Australia are summarised in Table 1. The waters collected from the springs were brackish Na-Cl-SO_4_-HCO_3_-type waters. The temperature of spring waters varied between 19.4 °C and 30.4 °C and electrical conductivity (EC) ranged between 2.5 and 7.9 mS/cm. the Oxidation/Reduction Potential (ORP) indicated that with the exception of Blanche Cup and The Bubbler, spring waters were generally reducing in character upon emergence. All alkalinities were greater than 400 mg/L (as CaCO_3_) and the pH of spring water was generally near-neutral on emergence (6.9 < pH < 7.4), apart from Beresford Spring (pH = 7.9). The partial pressure of CO_2_ (P_CO2_) values for all emergent spring water were several orders of magnitude greater than average atmospheric values, and the saturation index for calcite (SI_c_) ranged between −0.46 < SI_c_ < 0.76. Although the water composition varies between springs, the Ca^2+^ concentration is inversely correlated to alkalinity, and this relationship is controlled by the subsurface equilibrium of the water with calcite at a partial CO_2_ gas pressure of P_CO2_ = 10^−1.5^ atm (Fig. 4).

**Table 1:** Characteristics of mound spring study sites.

Spring	Abr.	Surface area of modern wetland m^2^	Surface area mound structure base m^2^	Total water-loss mm/day	Estimated loss: E.T. mm/day	Estimated loss: Infiltration mm/day
Beresford	B	840	2,308	6.2	2.0	4.1
Warburton	W	16,000	6,713	16.2	5.3	10.9
The Bubbler	TB	38,500	4,960	22.4	7.4	15.0
Blanche Cup	BC	1,400	3,023	15.4	5.1	10.3
Billa Kalina	BK	930	471	19.5	6.4	13.1

By virtue of this common control on the composition of the spring waters, a relationship between *t*_*c*_ and the spring water Ca^2+^ concentration exists that applies to the springs examined. By varying the Ca^2+^ concentration (*m*_*Ca*_) in the initial solution between 7.92 × 10^−4^ <*m*_*Ca*2_+<3.15 × 10^−3^ mol/kg H_2_O, a relationship between the initial Ca^2+^ concentration and the *t*_*c*_ was defined by regression analyses, and the following dependence between *t*_*c*_ and *m*_*Ca2+*_ was thus obtained (*R²* = 0.998):

1 $${m}_{Ca}=-\,2.2316397{\ast 10}^{-14}\ast {{t}_{c}}^{2}+1.9360257{\ast 10}^{-08}\ast {t}_{c}-9.5656106{\ast 10}^{-04}$$

**Figure 5:**
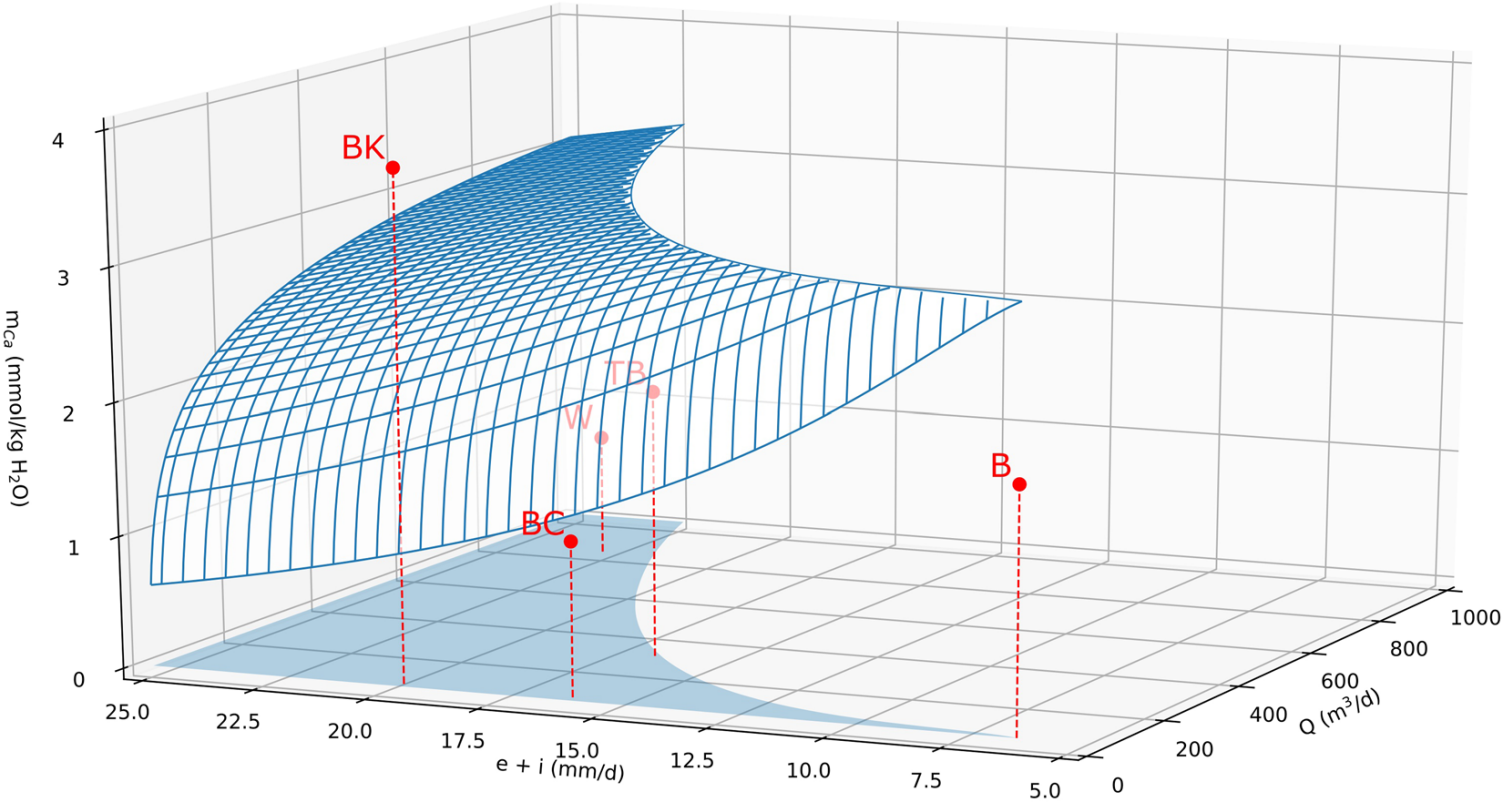
Three-dimensional plot displaying the surface where the characteristic timescale *t*_*w*_ and *t*_*c*_ are equal depending on the spring flow rate (*Q*), spring-water Ca^2+^ concentration and the water loss in the spring wetland due to evaporation (*e*) and infiltration (*i*). Four of the five springs (BC, B, W and TB) plot below this surface, indicating that their mound size is primarily controlled by the availability of lattice ions. The fifth spring (BK) plots above the surface, indicating its mound size is limited by water loss.

Equation (1) was used to draw the curved surface in Fig. 5, which marks where *t*_*w*_ and *t*_*c*_ are equal. Springs were classified as either water limited or lattice-ion limited based on their discharge and water loss rate (which determine *t*_*w*_) and Ca^2+^ concentration (which controls *t*_*c*_). The data points for all but one of the five springs plot below this surface, indicating that lattice-ion availability is limiting mound size. Billa Kalina spring is the only spring that falls in the water-limited regime.

**Figure 6:**
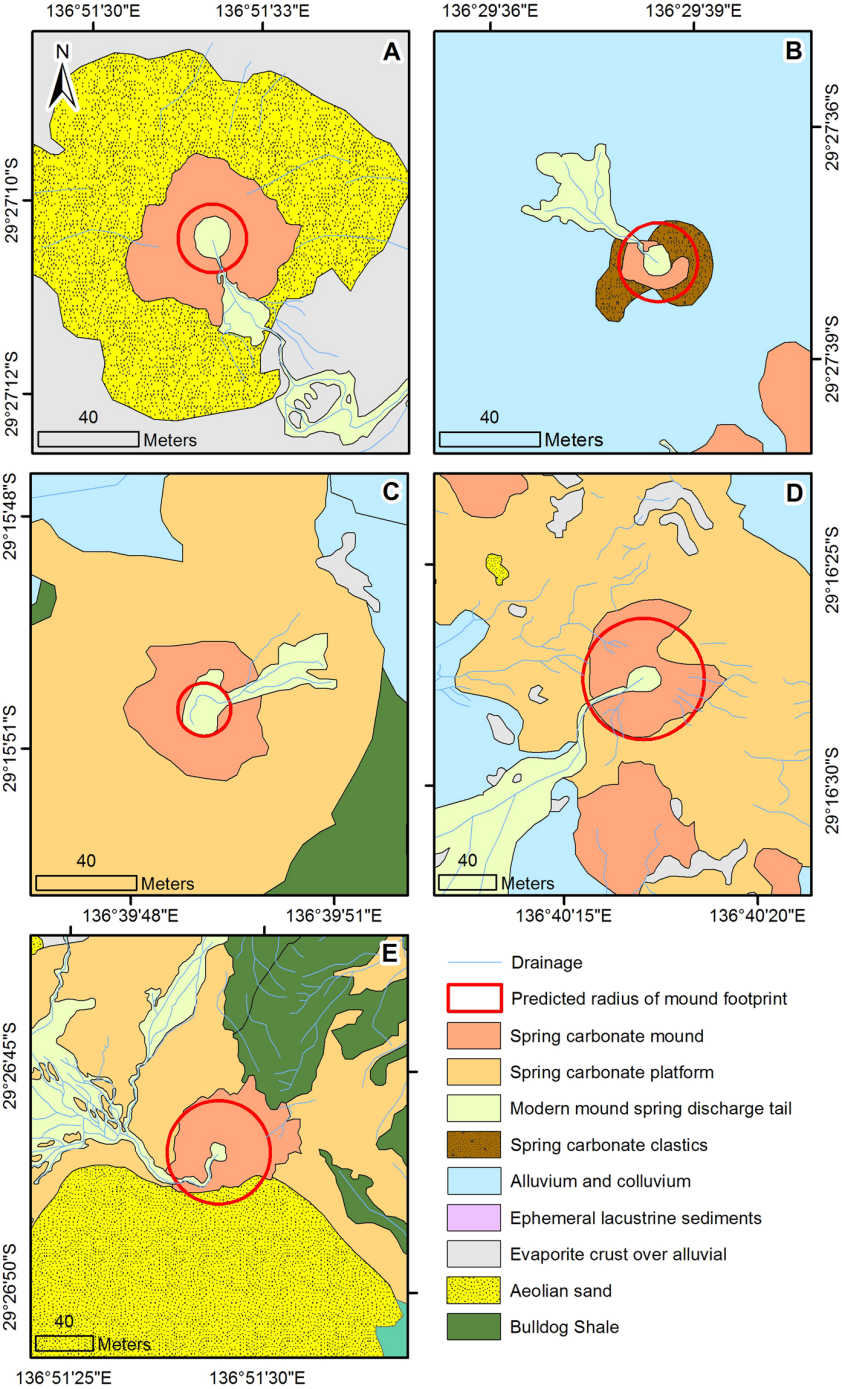
The simulated radius of carbonate precipitation for the five springs super-imposed on a map of the geological and morphological features of the mound spring environment. Study sites represented are (**A**) Blanche Cup (**B**) Billa Kalina Spring and (**C**) Beresford Spring, (**D**) Warburton Spring and (**E**) The Bubbler.

**Figure 7:**
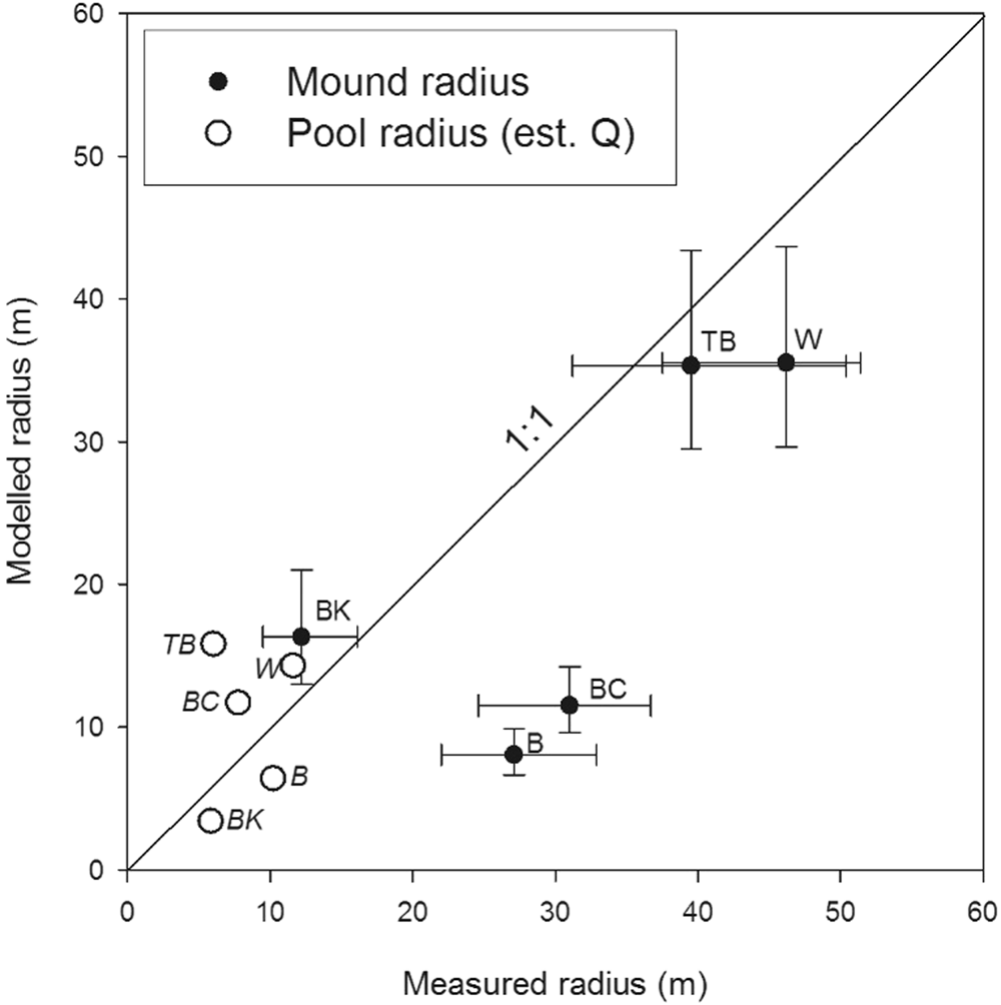
Scatter plot of the effective observed radius of each mound and pool structure versus the modelled radius of carbonate precipitation (solid black circles) and the calculated radius at which the maximum carbonate precipitation rate occurs (open circles). Vertical error bars represent the range of modelled radii based on +/−50% variations in *Q*. Horizontal error bars represent the difference between the minimum and maximum radii measured from the mound structures compared to the estimated average calculated radius (Table 1). For spring name abbreviations, see caption of Fig. 4.

The simulated radius is compared to the mapped geological and morphological spring mound for each site in Fig. 6. Without calibration, the model adequately approximates the observed radius for three of the five springs (Fig. 7, Table 2). Another metric calculated by the model is the distance from the vent at which the maximum carbonate mineral precipitation rate occurs. At this point, the vertical growth of the mound is fastest, which ultimately results in the build-up of a rim behind which the central pool forms. Comparison of this distance with the size of the modern pool indeed shows some agreement of the model with the field data (Fig. 7). The vertical carbonate growth rates calculated by the model are in the order of millimetres per year, which is consistent with previously published experimental work^12^, as well as hydrochemical mass balance calculations^15^.

**Table 2:** Discharge, average water depth, measured and modelled mound radii data.

Spring	*Abbreviations*	*Water flow (Q)* (L/s)	Average water depth (*h*_*c*_) (m)	Effective measured mound radius (m)	Modelled mound radius (m)	Discharge required for measured radius (L/s)
Beresford	B	0.06	0.016	27.1	8.2	1.5
Warburton	W	3.0	0.046	46.2	36.2	6.0
The Bubbler	TB	9.2	0.061	39.7	35.3	12.7
Blanche Cup	BC	0.25	0.023	31.0	11.5	3.4
Billa Kalina	BK	0.21	0.022	12.2	16.3	0.13

**Figure 8:**
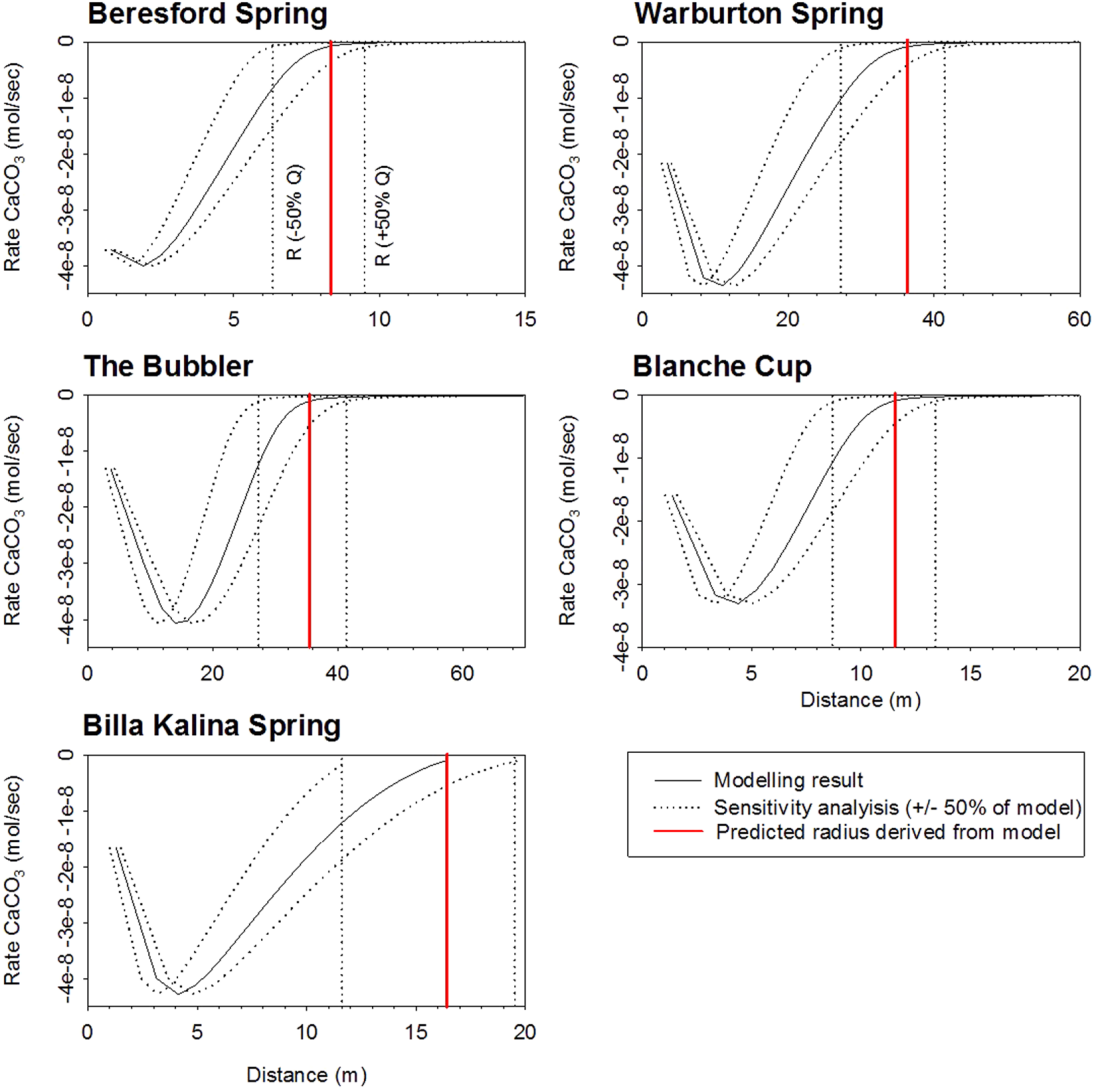
Graphs of modelled CaCO_3_ precipitation rates for spring examples. The model-calculated mound footprint radii are drawn as red lines where the calcite deposition rate <1 × 10^−10^ mol/kg H_2_O/s Dotted lines represent +/− 50% variations to discharge.

## Discussion

Our findings significantly reveal that carbonate mound morphology is not only dependent on the spring’s physical hydrological characteristics, but also, quantifiably, on the chemical composition of the emerging water. It explains why there is no correlation between these mound sizes and spring discharge; mounds of a high-discharge spring like The Bubbler remain relatively small as, due to the emerging water’s chemical composition, fast precipitation kinetics ensures that most carbonate precipitation occurs close to the vent. The calculated vertical growth rates of a few mm/yr imply that the time required for mound formation is in the order of centuries to a few millennia^12^, which is short relative to the age of many mound springs^16^. Nearly all mounds in the Kati Thanda-Lake Eyre South region are mature, thus it seems that the birth of new springs is infrequent at this period in geological time.

The model significantly underestimates the mapped radius of two of the four lattice-ion-limited mounds (Blanche Cup and Beresford), which are out by a factor of 3. This finding was expected though, as the present-day spring discharge used in the model is not necessarily representative for the discharge at the time of initial formation. In the Kati Thanda-Lake Eyre South region, spring discharge is believed to decrease with time^12,17^ due to increasing mound height^14,15,17^, decreasing groundwater pressures caused by abstraction or long-term climate-related recharge rates^12^, and/or vent blockage^13^. By attributing the differences in the simulated and observed radii to a decrease in spring flow rates, the model can be used to provide an estimate of the spring discharge at the initial stage of a mound’s formation by adjusting the discharge to match the modelled and measured radius (Fig. 7). The resulting discharges (Table 2) remain well within the range of discharges of other springs in the region^18^. An increase of the present-day discharge by a factor of 25 is required to increase the simulated size of the Beresford spring mound to attain the observed size.

The number of studied springs was necessarily limited in our study as only five sites had adequate aerial photographic coverage, minor disturbance from pastoralism and reliable recent discharge and hydrochemistry data in this remote region. Nonetheless, by virtue of the well-defined geometry of the spring mounds, our work clearly unveils the relationships between spring water chemistry, spring flow rate and wetland hydrology. Our findings equally apply to more complex carbonate deposits in which these fundamental controls are also expected to exist but are more difficult to recognise. For instance, similar-looking structures have been identified on Mars and their manifest morphological similarity has led to comparable hypotheses concerning formation via groundwater discharge^19,20^. Ancient martian hot springs have also been studied in Gusev crater by the *Spirit* rover mission^21^, strengthening the likelihood of spring mounds on that planet.

The quantification of the size metrics of the mound structures by reactive transport modelling was consistent with field observations, while deviations are attributable to the known decline of the spring flow rates with time. This demonstrates the value of the methodology in conceptual model development and validation, in palaeo-environmental studies based on carbonate archives or in hydrological studies. Finally, its potential application, with appropriate modification, in remote areas and possibly even extra-terrestrial hydrology^19–22^ where observations are largely limited to remotely-sensed data, potentially involving fluids other than water, is an exciting prospect.

## Methods

Spring mounds at five locations within the remote Kati Thanda-Lake Eyre South region of South Australia were analysed as part of this study. The spatial extent of the spring mounds and the features of the surrounding terrain were mapped using stereoscopic pairs of aerial photographs. The exception to this was Billa Kalina Spring, where only a single image was available; however, the prominence of the mound compared to the surrounding landscape at this site made it clearly identifiable. All interpretations were verified in the field by tracing the perimeter with a GPS device. Surface area was determined using GIS software, and the effective radius of each mapped mound was calculated as if the surface area was circular.

A table of water quality measurements and chemical analyses collected for each of the spring localities in 2008 and 2009 is provided as Table 3. The field methods used to obtain water samples have been published previously^13^.

**Table 3:** Water quality and chemistry results for the spring sites included in this study. All samples were taken from the spring vent.

Name	Date	Alk. CaCO_3_	Temp °C	Sp. EC mS/cm	pH	ORP mV	F^−^	Cl^−^	Br^−^	SO_4_^2−^	Ca^2+^	K^+^	Mg^2+^	Na^+^	Si^4+^	Sr^2+^	PCO_2_ matm	SI_C_
Beresford	Oct 08	580	20.7	6.56	7.87	−10.0	1.20	1558	1.73	262	74.2	46.1	37.3	1335	7.56	2.51	7.4	0.76
Warburton	Mar 09	546	27.3	6.05	7.06	−108.7	1.50	1470	1.60	277	80.1	43.3	38.4	1356	8.11	2.65	51.3	0.05
The Bubbler	Jul 09	814	30.4	2.46	7.15	4.9	2.90	1204	2.80	127	36.7	32.7	28.3	1160	6.09	1.36	66.1	0.05
Blanche Cup	Apr 11	681	23.5	6.87	7.21	67.7	1.7	1900	1.90	330	46.7	29.9	37.1	1310	4.71	2.17	42.66	−0.46
Billa Kalina	Mar 09	416	23.9	7.88	6.88	−138.9	0.34	2134	2.26	379	152.6	55.7	30.4	1745	5.49	4.18	53.7	−0.05

^a^All analytical determinations in mg/L unless otherwise stated.

The characteristic timescale for water flow, *t*_*w*_, is defined as:

2 $${t}_{w}={\int }_{0}^{0.95R}\frac{dr}{{v}_{w}}$$

where *r* is the radial distance from the spring vent (m), *R* is the maximum radial extent of the wetland (m), *v*_*w*_ is the water flow velocity (m/s), which decreases with increasing *r*, and *t*_*w*_ denotes the time it takes for the water to reach a distance of 0.95 *R*.

Steady-state conditions were assumed, i.e., the volumetric discharge rate of the spring *Q* (m^3^/s) equals the total rate of water lost via evapotranspiration plus the infiltration over the surface area *A* of the circular wetland. The value of *v*_*w*_ as a function of *r* then follows from:

3 $${v}_{w}(r)=\frac{Q-{\rm{\pi }}(e+i){r}^{2}}{2\pi {h}_{c}r}$$

where *h*_*c*_ is the wetland water depth, *e* is the evapotranspiration flux (m/s), and *i* the water infiltration rate per unit of wetland area (m/s). The water-loss rates *e* and *i* were assumed to be spatially uniform and represent an average between warmer and cooler months.

In order to determine *h*_*c*_, analogous to modern spring environments in the area, it was assumed that after initial mound formation, the resultant wetland was colonised by dense sedge and reed vegetation quickly compared to the time estimated to build up the mound^15^. Flow within a shallow, vegetated wetland may be described as slow and gravitationally controlled^23,24^. The following equation was used to model the friction effects of vegetation and detritus on flow in the wetland^23–26^:

4 $$q={K}_{w}{h}_{r}^{b}{S}^{a}$$

where *q* is discharge per unit width (m^2^/s), *K*_*w*_ is the hydraulic conductance coefficient for overland flow (m^2−b^/s), *h*_*r*_ is the height of the surface water column at radius *r* (m), *b* is an exponent related to vegetation microtopography, stem depth and density distribution, *S* is the hydraulic gradient (d*h*_*r*_*/*d*r*), *a* is an exponent that expresses the degree of flow turbulence^26^. Values of 1 and 3 for the exponents *a* and *b*, and a *K*_*w*_ value of 1.16 × 10^2^/m/s were adopted based upon previously published field-based experiments and subsequent recommendations for the modelling of wetlands with laminar flow and dense vegetation characteristics, such as those observed in mound spring wetlands^23,25,27^. Solving equation (4) for *h*_*r*_ subject to the following boundary conditions:

5 $$2\pi rq{|}_{r0}=Q$$

6 $$Q{|}_{R}={h}_{r}{|}_{R}=0$$

yields:

7 $${{h}_{r}}^{b+1}=\frac{b+1}{{K}_{w}}[\frac{Q}{2\pi }ln\frac{R}{r}-(e+i)(\frac{{R}^{2}-{r}_{0}^{2}}{4})]$$

Equation (7) was numerically integrated with respect to *r* and the result was divided by *R* to find the average water depth (*h*_*c*_) of the wetland that was used for subsequent calculations with Equation (3).

Discharge data to constrain *Q* were obtained from saline dilution tests or from temporary weir measurements over a series of field trips between October 2008 and July 2009. The evapotranspiration flux *e* and infiltration flux *i* were constrained using chloride and δ^18^O mass balance calculations^13^. Total water loss per unit surface area (*e* + *i*) was calculated by dividing the measured discharge by the area of the modern wetland environment, and was partitioned into rates for *e* and *i* using their relative proportions based on previously published data^13^. In this study, 33% of water loss was attributed to evapotranspiration and 67% to infiltration, which represents the average between results from warmer and cooler months.

Based on the reactive transport model by Keppel *et al*.^13^ for modern spring environments, the changing chemical composition of the water as it flows away from the spring vent was conceptualised to be due to: (i) CO_2_ degassing, (ii) evapotranspiration, and (iii) CaCO_3_ precipitation or dissolution. PHREEQC-2^28^ was used for modelling. This model was modified to reflect the flow conditions of the earliest stage of a spring, i.e., its initial surface manifestation.

The loss of dissolved CO_2_ to the atmosphere by degassing was described using the following rate expression^29^:

8 $$r{c}_{T}=k(C{O}_{2}-C{O}_{2(eq)})$$

where *rc*_*T*_ is the CO_2_ degassing rate (mol/kg H_2_O/s), *k* is the CO_2_ gas exchange rate constant (1/s), CO_*2*_ (mol/kg H_2_O) is the time-dependent aqueous CO_2_ concentration and *CO*_*2(eq)*_ (mol/kg H_2_O) is the aqueous CO_2_ concentration in equilibrium with atmospheric CO_2_ gas. A *k* value of 4.5 × 10^−4^/s was used in all examples and represents an average of the values previously adopted by Keppel *et al*.^13^ for the model of the modern spring environment. This value is also within the range of published values for stream environments^30–32^. Carbonate precipitation was based on the rate expression for calcite dissolution by using the kinetic rate equation by Plummer *et al*.^33^, which is implemented in the WATEQ. 4 F database^34^ of PHREEQC-2.

The modelling to quantify the timescale *t*_*c*_ was conducted using PHREEQC-2 by simulating CO_2_ degassing and kinetically-controlled carbonate precipitation in a batch-type model. Initial solutions were defined by equilibrating the solution with calcite and CO_2_ gas at a partial pressure of 10^−1.1^ atm. The latter value was chosen based on the relationship between alkalinity and the concentration of Ca^2+^ according to the field data (Fig. 4). Sodium (4.688 × 10^−2^ < *m*_*Na*_ < 6.416 × 10^−2^ mol/kg H_2_O, concentration *m*_*Na*_ was varied to achieve charge balance) and Cl^−^ (5.093 × 10^−2^ mol/kg H_2_O) were included as background electrolytes to obtain the approximate ionic strength of the spring waters. Calcite precipitation was considered to be negligible when the calculated precipitation rate dropped below 10^−10^ mol/kg H_2_O/s, which is roughly 3 orders of magnitude lower than the maximum rate found in the simulations.

PHREEQC-2 uses a mixing-cell approach to simulate transport along a flow-line, and the flow velocity is defined implicitly based on a fixed transport time step length and the length of a grid cell. Cell lengths were calculated by substituting *v*_*r*_ = *dr/dt* into Equation (3) and integrating the resulting expression, which yields:

9 $${t}_{n+1}-{t}_{n}=\frac{\beta ln(Q-{{r}_{n}}^{2})}{2\alpha }-\frac{\beta ln(Q-{{r}_{n+1}}^{2})}{2\alpha }$$

In which *n* denotes the cell number, $\alpha =\pi (e+i)$ and *β* = 2*πh*_*c*_. Solving for *r*_*n*+1_, gives:

10 $${r}_{n+1}=\sqrt{\frac{Q-(Q-\alpha {{r}_{n}}^{2})\exp (\frac{2\alpha }{\beta }({t}_{n}-{t}_{n+1}))}{\alpha }}$$

By setting a time step (*t*_*n*_*−t*_*n*+1_) and assigning a radial distance (*r*_1_) for the first cell, equation (10) was used to calculate the lengths of the consecutive cells. The one-dimensional discretisation thus varied for each of the five springs modelled, depending on their flow rate and the evapotranspiration and infiltration fluxes (Table 1). The size of the mound was taken as the distance along the flow path where the calcite precipitation rate fell below 10^−10^ mol/kg H_2_O/s, which is less than 0.5% of the maximum rate observed (Fig. 8).
